# Treatment of Chronic Kidney Disease with Extracellular Vesicles from Mesenchymal Stem Cells and CD133^+^ Expanded Cells: A Comparative Preclinical Analysis

**DOI:** 10.3390/ijms23052521

**Published:** 2022-02-25

**Authors:** Dayane Mayumi Miyasaki, Alexandra Cristina Senegaglia, Sérgio Adriane Bezerra de Moura, Amanda Leitolis, Luiz Guilherme Achcar Capriglione, Letícia Fracaro, Lidiane Maria Boldrini Leite, Paulo Henrique Utumi, Felipe Yukio Ishikawa Fragoso, Fernando Meyer, Alejandro Correa, Paulo Roberto Slud Brofman

**Affiliations:** 1 Core for Cell Technology, School of Medicine, Pontifícia Universidade Católica do Paraná (PUCPR), Curitiba 80215-901, Brazil; miyasaki.dayane@gmail.com (D.M.M.); acsenegaglia@hotmail.com (A.C.S.); luizcapriglione@yahoo.com.br (L.G.A.C.); leticiafracaro@gmail.com (L.F.); lidiane.leite@pucpr.br (L.M.B.L.); utumipr@hotmail.com (P.H.U.); yukio.ishikawa@gmail.com (F.Y.I.F.); fmeyer@urocentro.com (F.M.); paulo.brofman@pucpr.br (P.R.S.B.); 2 INCT—REGENERA National Institute of Science and Technology in Regenerative Medicine, Rio de Janeiro 21941-902, Brazil; aleitolis@gmail.com; 3 Department of Morphology, Campus Universitário Lagoa Nova, Universidade Federal do Rio Grande do Norte (UFRN), Natal 59072-970, Brazil; sergioabm@gmail.com; 4 Laboratory of Basic Biology of Stem Cells, Carlos Chagas Institute, Fiocruz-PR, Curitiba 81350-010, Brazil

**Keywords:** chronic kidney disease, extracellular vesicles, mesenchymal stem cell, endothelial progenitor cell, adenine

## Abstract

Chronic kidney disease (CKD) is characterized by structural abnormalities and the progressive loss of kidney function. Extracellular vesicles (EVs) from human umbilical cord tissue (hUCT)-derived mesenchymal stem cells (MSCs) and expanded human umbilical cord blood (hUCB)-derived CD133^+^ cells (eCD133^+^) maintain the characteristics of the parent cells, providing a new form of cell-free treatment. We evaluated the effects of EVs from hUCT-derived MSCs and hUCB-derived CD133^+^ cells on rats with CDK induced by an adenine-enriched diet. EVs were isolated by ultracentrifugation and characterized by nanoparticle tracking analysis (NTA) and electron microscopy. The animals were randomized and divided into the MSC-EV group, eEPC-EV group and control group. Infusions occurred on the seventh and 14th days after CKD induction. Evaluations of kidney function were carried out by biochemical and histological analyses. Intense labeling of the α-SMA protein was observed when comparing the control with MSC-EVs. In both groups treated with EVs, a significant increase in serum albumin was observed, and the increase in cystatin C was inhibited. The results indicated improvements in renal function in CKD, demonstrating the therapeutic potential of EVs derived from MSCs and eCD133^+^ cells and suggesting the possibility that in the future, more than one type of EV will be used concurrently.

## 1. Introduction

Chronic kidney disease (CKD) is characterized by structural abnormalities and the progressive loss of kidney function and is prevalent and considered a global health problem [1]. In recent decades, the prevalence of CKD has increased and contributed to reductions in quality of life, mortality, and substantial health care costs [2,3]. The progressive deterioration observed in CKD is the consequence of a series of inflammatory events that lead to tubular atrophy, glomerulosclerosis, interstitial cell infiltration, and fibrosis [4]. The treatments for CKD consist of reducing the lesion, delaying disease progression, and renal replacement through kidney transplantation or dialysis, and although these are procedures that have been performed for many years, there are still risks related to these procedures, therefore, during the last decades, research to find new therapeutic strategies in repairing renal damage in CKD is essential. New therapeutic strategies for tissue repair have emerged, and one of the most promising approaches is the use of human mesenchymal stem cells (MSCs), endothelial progenitor cells (EPCs) CD133^+^ and extracellular vesicles (EVs) derived from these cells [5,6,7].

Studies have already shown that MSCs and CD133^+^ cells help to regenerate damaged tissue. MSCs act in several processes, preventing injuries and promoting renal recovery by reducing apoptosis, suppressing oxidative stress, and immunomodulation, among others. These beneficial effects are achieved through paracrine actions, the secretion of growth factors and cytokines, and the release of EVs [5,8,9,10]. CD133^+^ cells are precursor cells that are capable of differentiating into mature endothelial cells and contribute to the formation of new blood vessels.

This unique cell population shares characteristics with hematopoietic cells and is incorporated at physiological or pathological neovascularization sites in response to angiogenic growth factors. EVs derived from stem cells have beneficial and protective effects and are involved in processes such as immunomodulation, hemostasis, vessel integrity, and tissue regeneration and affect the development and function of organs and systems, including the kidney [8,11]. EVs are involved in most of the physiological processes associated with intercellular communication. These processes, such as immunomodulation, hemostasis, vessel integrity and tissue regeneration, affect the development and function of organs and systems, including the kidney [6]. Due to the various signals carried by these vesicles through the horizontal transfer of functional biomolecules, the effects of vesicles derived from MSCs and EPCs cells were tested on different models of renal, cardiologic and lung diseases and were shown to repair kidney, heart and lung tissue after injury [12,13,14,15]. EVs have great therapeutic potential since they maintain the characteristics of their parent cell and represent a new cell-free therapeutic strategy. We evaluated the effects of EVs (exosomes and microvesicles) from human umbilical cord tissue (hUCT)-derived mesenchymal stem cells (MSCs) and expanded human umbilical cord blood (hUCB)-derived CD133^+^ cells (eCD133^+^) on 30 Wistar rats with CKD induced by excess dietary adenine (ADE).

The chronic kidney disease model used in this study was based on an adenine-enriched diet, a well-established model that was first used by Yokozawa et al., 1982 [16]. Some studies have focused on the use of EVs as treatments for CKD, but none to date have compared EVs derived from different sources (MSCs and expanded human umbilical cord blood (hUCB)-derived CD133^+^ cells (eCD133^+^)) under the same conditions. Since MSCs and eCD133^+^ cells act on different pathways to repair injuries, the use of EVs from both cell types in this study becomes important, allowing for better understanding of the pathways of action of cells and allowing researchers to choose the best source for each treatment.

## 2. Results

### 2.1. Cell Expansion and Characterization

**Figure 1:**
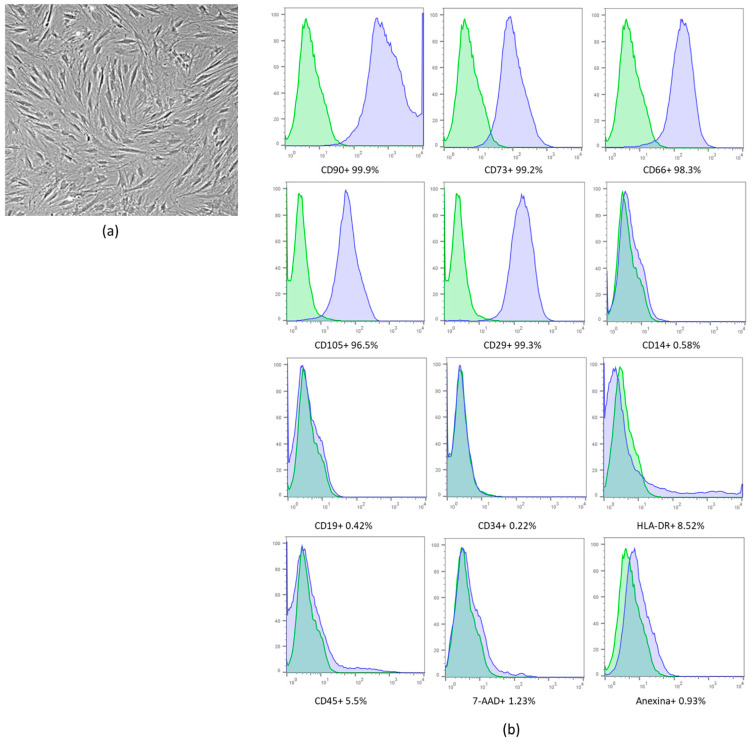
(**a**) MSCs after expansion. (**b**) Immunophenotypic analysis by flow cytometry. Umbilical cord blood-derived mesenchymal stromal cell cord (MSCs-hUCT) characterization (representative set from one donor). The blue histograms indicate the percentage of the population that was positive for each antibody, while the green histograms indicate the isotype controls. For 7-AAD analysis and autologous control was used. Scale bar: 500 µm. The results are expressed as percentages (%).

After the isolation and expansion of cells (Figure 1a), 22 × 10^6^ MSCs-hUCT were obtained. Immunophenotypic characterization of MSCs was performed according to the ISCT criteria and showed that the cells were positive for CD90, CD73, CD166, CD105 and CD29 and negative or reduced for CD45, CD14, CD19, CD34 and HLA-DR. The 7-AAD and Annexin V assay results demonstrated that the cells were viable (Figure 1b).

**Figure 2:**
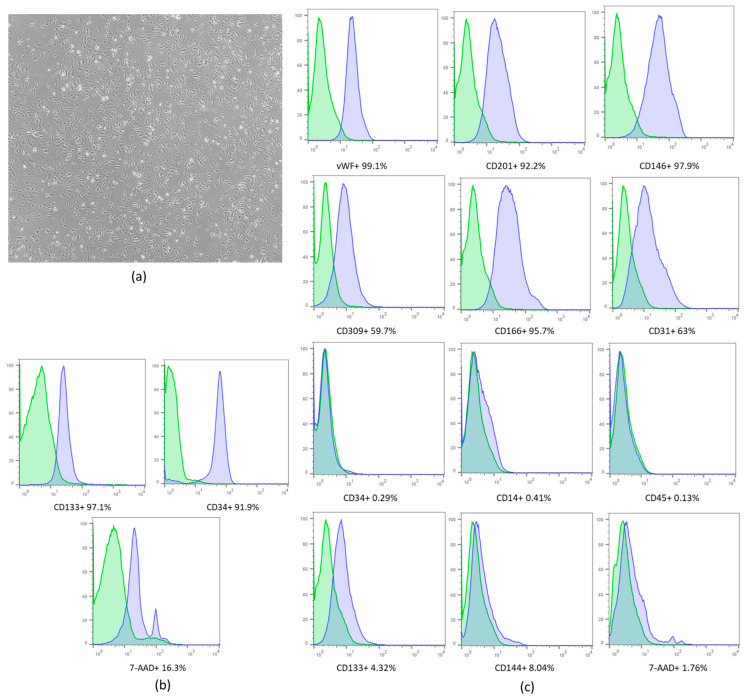
(**a**) Culture of EPCs. Immunophenotypic analysis by flow cytometry. (**b**) Purified CD133^+^ cell characterization (representative set from one donor). (**c**) Expanded CD133^+^ cell characterization. The blue histograms indicate the percentage of the population that was positive for each antibody, while the green histograms indicate the isotype controls. For 7-AAD analysis and autologous control was used. Microscope objective: 5×. The results are expressed as percentages (%).

After the expansion (Figure 2a) and isolation of hUCB cells, the average number obtained was 68 × 10^6^ mononuclear cells (MCs). After purification and expansion, 77 × 10^6^ cells were obtained. Immunophenotypic characterization of CD133^+^ cells was performed after expansion and showed low expression levels of CD133 and CD 34 compared to the immunophenotypic profile of purified CD133^+^ (Figure 2b). High expression of endothelial cell markers such as the von Willebrand factor (vWF) and CD146 was observed (Figure 2c). This profile clearly shows that the phenotype of the expanded CD133^+^ cells, as determined by the surface markers, changed after expansion, and the cells acquired an endothelial-like phenotype.

### 2.2. Isolation and Characterization Extracellular Vesicles

**Figure 3:**
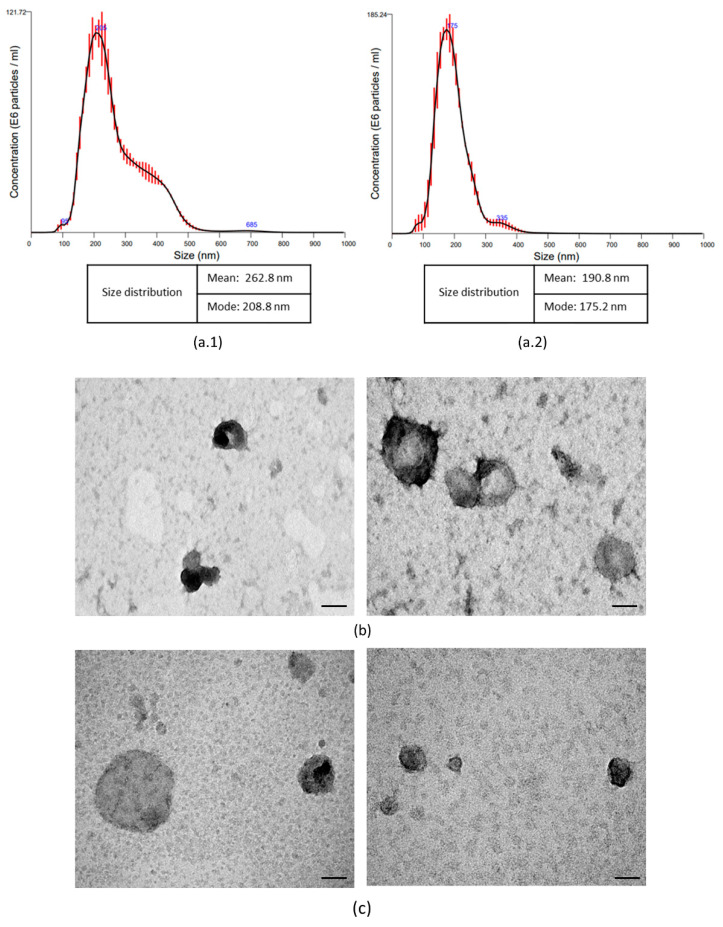
Characterization of extracellular vesicles derived from MSCs and expanded CD133^+^ cells. Graph A represents the distribution of extracellular vesicle sizes, as determined by NTA. (**a.1**) The size distribution of EVs derived from MSCs, and (**a.2**) the size distribution of expanded CD133^+^ cells. (**b**) Representative transmission electron microscopy (TEM) image of EVs derived from MSCs hUCT and (**c**) representative TEM image of extracellular vesicles derived from CD133^+^ hUCB-derived. Scale bar: 200 µm.

The number of EVs recovered from 22 × 10^6^ MSCs was 10.2 × 10^11^ particles, with an average particle size in the 262.8 nm range. The most frequent diameter of vesicles derived from MSCs was 208.8 nm. The total number of EVs recovered from 77 × 10^6^ eCD133^+^ cells was 80.64 × 10^9^ particles, with an average particle size in the 190.8 nm range. The most frequent diameter of vesicles derived from eEPCs was 175.2 nm (Figure 3).

As determined by the transmission electron microscopy (TEM), EVs isolated from both sources showed rounded shapes and diameters within the size range calculated by nanoparticle tracking analysis (NTA). Additionally, the presence of double membranes was identified in some particles. NTA and TEM suggested that the vesicle samples had satisfactory purity and quality. EVs that had been previously isolated from MSCs and eCD133^+^ (same donor) had been previously analyzed by immunogold labeling/TEM by our group [17].

### 2.3. The Animal Model Showed the Characteristics of CKD

Adenine administration induced renal damage and significantly increased the levels of serum creatinine (Cr) (*p* = 0.001) and cystatin C (Cys C) (*p* = 0.001) compared to preinduction times and at day seven after CKD induction (Table 1). Although there were no significant differences for the biomarker albumin (*p* = 0.061), a decreasing trend in this serum marker was observed (Table 1).

### 2.4. Treatment with EVs and Biochemical Analyses

**Figure 4:**
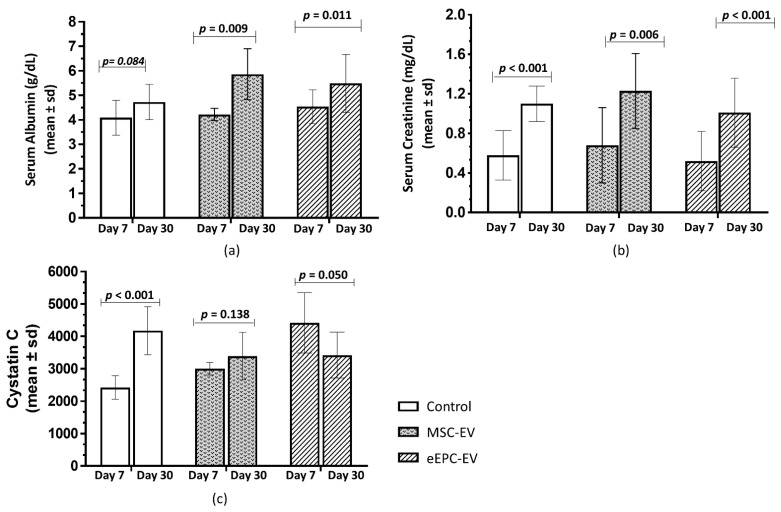
Serum albumin, creatinine and cystatin C evaluation. Analysis was performed on the 7th day after CKD induction by adenine and on the 30th day after infusion with EVs. (**a**) There was a significant difference in serum albumin levels between the MSC-EV and eEPC-EV groups. (**b**) Significant increase in serum creatinine in all the groups. (**c**) Significant increase in cystatin C levels in the control group. The data in are reported as the mean ± sd. *p* < 0.05 is considered significant.

Serum evaluations were performed before the first infusion on the seventh day and after the two infusions on the 30th day to evaluate the effects of treatment with EVs derived from MSCs and eCD133^+^ cells. Serum albumin levels increased significantly in the MSC-EV and eEPC-EV groups (*p* = 0.009 and *p* = 0.011, respectively) and did not increase significantly in Group C (*p* = 0.084) (Figure 4a).

A significant increase in serum creatinine (C Group *p* < 0.001, MSC-EV Group *p* = 0.006 and eEPC-EV Group *p* < 0.001) was observed when comparing the values on the seventh and 30th days in all groups (Figure 4b). There was also a significant increase in serum cystatin C in Group C (*p* < 0.001), but there was no significant increase in the MSC-EV (*p* = 0.138) and eEPC-EV (*p* = 0.050) groups (Figure 4c).

### 2.5. Histological Analysis Showed the Induction of CKD and Improvements in the Disease after EV Treatment

**Figure 5:**
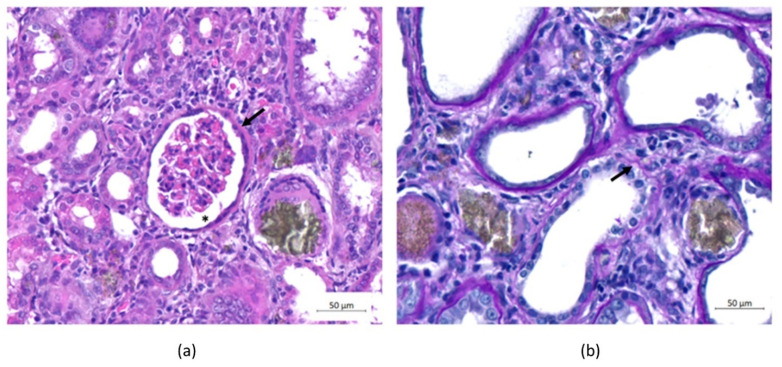
Histopathological evaluation of the kidneys. (**a**) Hematoxylin and eosin (H&E) staining and (**b**) Schiff’s periodic acid (PAS) staining. (**a**) The animals were evaluated on Day 30, and an increase in Bowman’s capsule thickness (arrow) and capsular space hypertrophy (asterisk) was observed in all groups. (**b**) Discontinuity of the basal blade (arrow) was observed in all groups. Scale bar: 50 µm.

Histological observations of renal sections from all animals with CKD revealed thickening of the Bowman’s capsule, hypertrophy of the capsular space and discontinuity of the basal lamina (Figure 5).

Orally administered adenine is immediately metabolized into 2,8-dihydroxyadenine (2,8-DHA), which precipitates and forms crystals in microvilli and the apical epithelial region of the proximal tubule, causing degenerative changes in the renal tubules and interstice, leading to renal failure in the final stage [18,19].

Dietary administration of the purine adenine is already a model for CKD induction that has been well established in the literature, and histological changes and 2,8-DHA crystal formation show the effectiveness of this CKD model.

**Figure 6:**
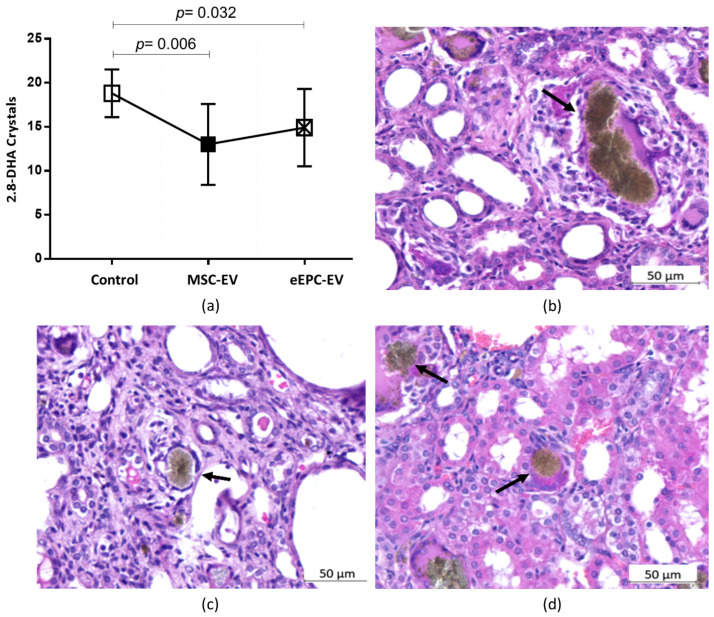
(**a**) Number of crystals present in renal tubules. There was a significant difference in the amount of crystal deposition between the groups (*p* = 0.014) and when comparing group C with the MSC-EV group (*p* = 0.006) and group C with the eEPC-EV group (*p* = 0.032). The data in the figure are the mean ± sd. *p* < 0.05 is considered significant. (**b**–**d**) Histopathological evaluation of the kidneys. H&E staining. Different dimensions of the crystals (arrows). The dimensions of the crystals in (**b**) (Group C) were more extensive than those observed in (**c**,**d**) (groups MSC-EV and eEPC-EV, respectively).

After treatment with extracellular vesicles, there was a significant difference in the amount of crystal deposition between the groups (*p* = 0.014). When evaluated two by two, there was a significant between Group C and the MSC-EV group (*p* = 0.006) and between Group C and the eEPC-EV group (*p* = 0.032), but there was no significant difference between the MSC-EV group and the eEPC-EV group (Figure 6a).

Qualitative analysis revealed that the size of the crystals in the renal tubules was different. In Group C, the crystals were undoubtedly more voluminous than those observed in groups MSC-EV and eEPC-EV (Figure 6b–d).

**Figure 7:**
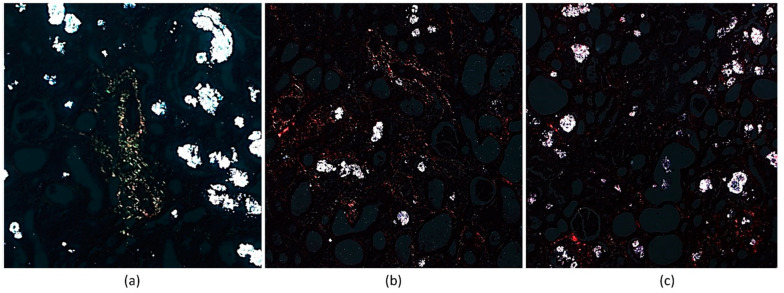
Picrosirius red stained (PRS) image of renal tissue. (**a**) MSC-EV, (**b**) eEPC-EV and (**c**) Control. It was possible to observe collagen fibers, stained red and green, in all groups analyzed. Microscope objective: 20×.

Using Picrosirius red staining, a sensitive method that identifies fibrillar collagen networks in the renal tissue, it was observed that the presence and heterogeneity of collagen fiber in all the analyzed groups occurred as a result of a fibrotic process (Figure 7).

**Figure 8:**
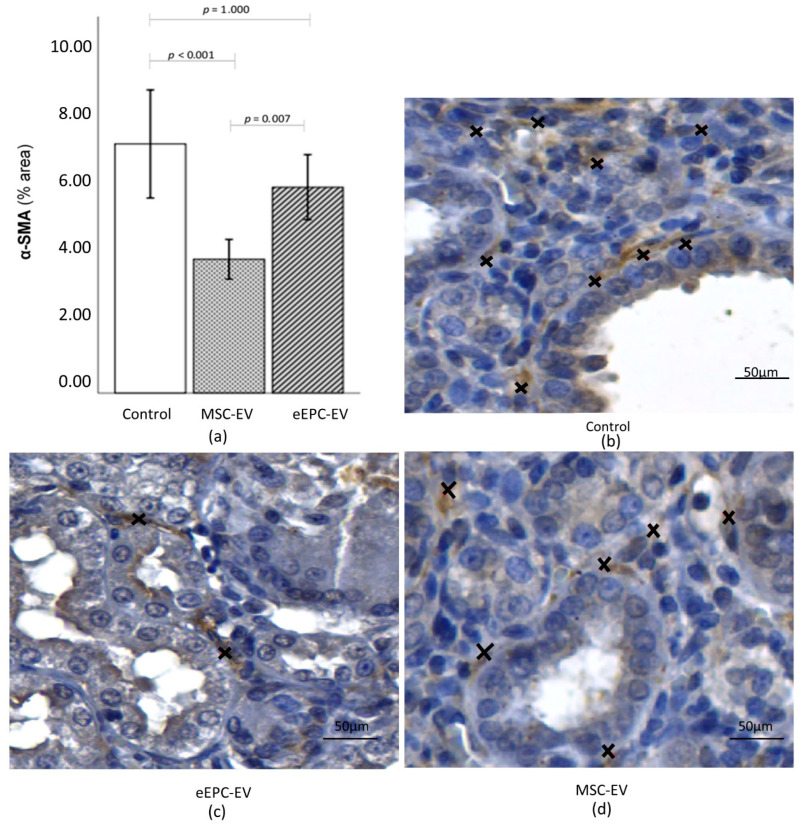
Immunohistochemical staining of α-SMA. (**a**) There was a significant difference in the expression of α-SMA when comparing the C Group and the MSC-EV group (*p* < 0.001) and MSC-EV and the eEPC-EV group (*p* = 0.007). The data in figure are the mean ± sd. *p* < 0.05 is considered significant. (**b**–**d**) α-SMA expression (x) in (**b**) (Group C) was higher than that observed in (**c**,**d**) (groups eEPC-EV and MSC-EV, respectively).

Immunohistochemical staining of alpha-smooth muscle actin (α-SMA) showed a significant increase in α-SMA expression between the C Group and MSC-EV group (*p* < 0.001) and between the MSC-EV and eEPC-EV group (*p* = 0.007), but there was no significant difference in α-SMA expression between the C Group and the eEPC-EV group (Figure 8).

## 3. Discussion

Comparison of the mechanisms of action of EVs from eCD133^+^ cells and MSCs in the treatment of CKD is critical because these two sources act differently in repairing injury. Knowing whether one of these two sources can have better results can help when choosing the best therapy or possibly combining the two. Studies have demonstrated the potential of MSCs and eCD133^+^ cells in treating acute or chronic heart disease and respiratory and renal diseases [20,21,22,23,24]. Studies suggest that the therapeutic effect of these cells is mediated by modulation of the immune system, paracrine factors and the release of EVs [25,26,27].

The number of EVs isolated from eCD133^+^ cells and MSCs was consistent with that presented in the literature and the characterization of EVs using MET and NTA, verifying the identity of EVs [17,28,29].

The adenine model has been used by many studies [30,31,32,33,34] to induce CKD and test several types of treatments, as this model causes metabolic alterations that reproduce CKD leading to tubulointerstitial fibrosis and, as a result, leads to end-stage renal disease. The studies also showed that this model altered urinary levels, increased creatinine levels, decreased creatinine clearance, and altered other plasmatic examinations performed. In our study, the adenine model of CKD led to a robust damage to renal functions, which were observed by serological analyses and severe histological alterations, compromising renal tissue as a result of inflammatory processes.

Serum albumin is often defined as an indicator of nutritional status, and in inflammatory conditions, it is eventually blocked [35]. After the induction of the disease, a decrease in serum albumin was expected. In our study, this decrease was not significant compared to the levels preinduction and seven days after the administration of adenine (prior to the infusion of EVs), possibly since CKD was still being established during this period. However, a decreasing trend in this marker was observed, and with a longer evaluation time, albumin levels may be further reduced due to the progression of CKD. In studies that used adenine for CKD induction, albumin levels were not evaluated, or the analyses were not performed during similar periods as in this work [18,36,37,38,39]. In addition, some factors influence serum albumin concentrations, such as changes in the distribution of body fluids, hydration conditions, body weight loss and the rates of synthesis and catabolism, which may affect the results obtained [40]. When evaluating albumin levels on the 30th day, there was a significant increase compared to that on the seventh day in the MSC-EV and eEPC-EV groups, maybe indicating disease stability; this increase was not observed in the control group, which did not receive treatment with EVs.

When the kidneys are not functioning properly, there is an increase in the levels of blood creatinine, which a product of muscle metabolism [41]. Serum Cr is the most common endogenous biomarker used to estimate the glomerular filtration rate (GFR). The increase in serum creatinine levels on the seventh day compared to the preinduction level indicates that there was an induction of CKD, which can be observed in some studies [30,31,33,42]. After treatment with EVs, a significant reduction in this marker was expected, but this outcome was not demonstrated. One reason is that serum Cr, as a marker of CKD, should be used with care because its concentrations may vary according to many factors, such as muscle mass, muscle metabolism, body weight, nutritional status, and physical activity, which may alter its levels [43,44,45,46,47].

Due to these creatinine limitations, a new, more accurate marker has been used, Cys C. Cys C is a cysteine protease inhibitor that is synthesized by all nucleated cells in the body and is freely filtered by the glomerulus and this filtration is fully catabolized in the proximal renal tubule and does not return to the blood, and unlike serum Cr, cystatin C is completely reabsorbed and not secreted [45]. When the GFR decreases, the cystatin C level begins to rise proportionally [48,49]. In this work, on the 30th day, Cys C did not increase significantly in the groups that received treatment with EVs; however, there was a significant increase in the control group. This result may suggest that there was improvement in renal function after treatment, but the use of other renal function markers in association with Cystatin C would be essential to confirm it.

Furthermore, there is evidence that serum Cystatin C concentrations may increase before serum Cr in acute kidney injury (AKI) but that Cys C concentrations decrease before creatinine in many hospitalized patients, being a biomarker of AKI recovery in hospitalized patients [50]. In our evaluations, Cys C levels decreased before creatinine concentrations, suggesting improvement or stability of CKD in the treated animals, as it has been shown that Cys C is a more accurate marker and can be used as an earlier outcome biomarker to serum Cr.

We observed crystal formation, Bowman’s capsule thickening, enlargement of the capsular space due to glomerular atrophy, basal membrane interruptions, and areas with fibrosis, which shows that the CKD model using adenine was effective [42,51,52]. There was the deposition of 2.8 DHA crystals along the renal tubules and an inflammatory reaction associated with a granulomatous reaction and the presence of multinucleated giant cells. However, the number of crystals was significantly increased in Group C, which did not receive any treatment. The crystals present in Group C were larger than those observed in the groups treated with EVs. Possibly, the quantity and larger size of the crystals in Group C indicates that there were increased inflammatory processes, since the granulomatous reactions were directly proportional to the quantity and size of the crystals. In the MSC-EV and eEPC-EV groups, the inflammatory process may have been reduced due to EV treatment and resulted in smaller and reduced amounts of crystal formation.

The first response to kidney injury is inflammation, which can lead to renal fibrosis [53]. Injury leads to the loss of renal tissue architecture and the release of inflammatory mediators, which induce the accumulation of mononuclear cells in the renal interstitium; macrophages and T lymphocytes are activated and release cytokines, promoting a phenotypic response in tubular epithelial and endothelial cells, causing apoptosis, mesenchymal epithelial transition or endothelial–mesenchymal transition to occur, transforming cells into interstitial fibroblasts [53,54,55].

Fibrosis is the final pathological process associated with maladaptive repair and is defined by extracellular matrix formation and accumulation [55]. Fibrosis can affect the kidney, lungs, heart, eyes and several organs and can be triggered by trauma, wounds, infection, metabolic disorders, autoimmunity, inflammation and other factors, which converge in the molecular signals responsible for initiating and driving fibrosis [56,57,58]. Fibrosis can be considered a disproportionately high and prolonged wound healing response.

In normal wound healing, myofibroblasts undergo apoptosis, but the reparative response ends when the damaged tissue has been repaired. In chronic progressive renal disease, fibroblasts in the kidney interstitium assume a contractile myofibroblastic phenotype and are responsible for the formation of the extracellular matrix that is rich in collagen and fibronectin during wound healing and at wound healing and fibrosis sites, consequently leading to a decline in renal function [59]. Myofibroblasts are involved in the development and progression of malignant tumors [60], in stimulating cancer cell growth and also activate the initiation of angiogenesis [61]. They also play a role in hepatic fibrosis and renal fibrosis [62,63]. Myofibroblasts are activated by a variety of mechanisms, including paracrine signals derived from lymphocytes and macrophages. Cytokines (IL-13, IL-21, TGF-β1), chemokines (MCP-1, MIP-1β), angiogenic factors (VEGF), growth factors (GF), caspases and components of the renin-angiotensin-aldosterone system (ANG II) have been identified as important regulators of fibrosis [64]. After renal injury, pro-fibrotic factors are secreted by injured tubular epithelia and infiltrating inflammatory cells, promoting signaling events that lead to myofibroblast activation, proliferation and production of extracellular matrix [65,66].

Macrophages are present at sites of active renal fibrosis that contain α-smooth muscle actin (α-SMA)-positive, matrix-producing myofibroblasts. The degree of macrophage infiltration correlates with both the severity of renal damage and the extent of renal fibrosis, thereby establishing a close relationship between the presence of macrophages and renal fibrosis in CKD [55,67]. In this context, we evaluated α-SMA expression to assess the effects of EVs on adenine-induced CKD since the protein expression of α-SMA correlated with the activation of myofibroblasts.

Studies [68,69] have shown that MSCs can reduce the expression of α-SMA, TGF-β1, collagens, and TNF-α in rat models of renal injury. Researchers [70,71,72] injected microvesicles derived from Wharton’s Jelly-derived MSCs (WJ-MSCs) into rats with kidney injuries, and there were clear improvements in renal function, decreased α-SMA and TGF-β1 expression and improved survival rates. In this work, we observed a significant increase in α-SMA expression in the control group compared to the MSC-EV group.

We observed a fibrotic process in the renal tissue in all groups; however, α-SMA labeling showed that there was a significant increase in this marker only in the control group. Alpha-SMA-positive myofibroblasts have been identified as the primary cell type responsible for the accumulation of interstitial matrix in fibrotic diseases [73]. Thus, the absence of a significant increase in α-SMA labeling in the treated groups may be an indication that there is slower or no progression of fibrosis in these groups.

MSCs and CD133^+^ cells have already been studied in regenerative medicine in many diseases. Several studies have shown the therapeutic potential of these cells in the treatment of acute [74,75,76] and chronic [77,78,79] kidney injuries, and it is believed that this therapeutic potential is due to the paracrine capacity of these cells, which secrete growth factors and cytokines for the regeneration process [80] and the release of EVs. It is evident that the paracrine mechanisms of these cells helps cell repair through EV secretion.

Due to the potential of EVs to be involved in many processes, including immunological signaling, angiogenesis, the stress response, senescence, cell proliferation and differentiation, EVs have been identified as a therapeutic tool for use in cell-free therapies due to their lower propensity to trigger innate and adaptive immune responses [81].

The literature has already demonstrated the regenerative potential of EVs derived from MSCs and eCD133^+^ cells in renal injury. In 2012, Bruno et al. [25] investigated the effects of EVs on the survival of mice with cisplatin-induced kidney injuries and showed that a single administration of EVs improved kidney function and morphology, in addition to improving survival. However, this treatment did not prevent chronic tubular damage or the persistent increase in urea and creatinine. However, multiple EV injections further decreased mortality, and surviving mice showed improvements in histology and renal function. The protective mechanism was mainly attributed to an antiapoptotic effect of EVs [25].

Cantaluppi et al., 2012 [13] tested the effects of EPC-EVs in preventing acute kidney injury in an ischemia–reperfusion injury model in rats and observed that EPC-EVs increased cell proliferation and reduced apoptosis in tubular epithelial cells and protected the kidney against the progression of chronic injury by transferring proangiogenic miRNAs to resident renal cells. Nassar et al., 2016 [82] administered two infusions of EVs derived from umbilical cord MSCs in patients with advanced-stage CKD. The researchers observed that MSC-derived EVs mimicked the beneficial effects of MSC administration, which led to an improved inflammatory immune response and transient (3–6 months) renal function. In general, as observed in these studies, EVs could promote functional and morphological recovery of the kidney, corroborating the results obtained in this work.

EVs promote cell-to-cell communication via the transfer of functionally relevant biomolecules and, therefore, can be harnessed for therapeutic purposes similar to their parent cells and can be readily isolated from many cell types, such as MSCs and EPCs. In a previous study by our group, proteins present in eCD133^+^ EVs (same samples used in this study) and human bone marrow MSC-EVs (hMSC-EVs) were identified by mass spectrometry [17]. Functional enrichment analysis of the shared proteins in the two EVs samples identified, with statistical significance, sets of proteins related to cell maintenance, cell cycle, and negative regulation of apoptosis, among others. EVs derived from these cells can provide molecules to regulate the redox state of target cells under oxidative stress. Kidney failure generates oxidative stress due to increased oxidant activity and reduced antioxidant capacity [83]. Specific proteins associated with protection against oxidative stress were identified in the vesicles, such as glutathione S-transferase (GST), protein disulfide isomerases (PDIA3, PDIA4 and PDIA6), lactate dehydrogenase A (LDHA) and B (LDHB) and superoxide dismutase (SOD) [17].

Furthermore, the transmembrane receptor Plexin-B2, essential for kidney repair [84], was also identified in both types of EVs. Angiogenesis-related proteins such as the von Willebrand factor (vWF) were overrepresented in eCD133^+^ EVs and proteins with cellular differentiation functions such as those involved in establishing planar polarity (Wnt signaling pathway) in hMSC-EVs. All these previous findings not only explain, at least partially, the improvements seen in the groups of animals treated with EV but may also support the combined use of these vesicles for CKD.

One limitation of the present study was finding a model that mimics CKD as closely as possible without high mortality rates by the end of the study. Adenine is a model for CKD successfully used for many years. However, this model has restrictions, as the intensity of the injury caused depends on the concentration of adenine offered in the diet. There was a significant injury to the kidney tissue in this study, which may have made the analysis difficult. The treatments would have possibly been more effective in a milder stage of CKD. Therefore, for future studies, we believe it could be necessary to use lower adenine concentrations or a different model of CKD. Another limitation of this study is the reduced amount of samples to perform other types of renal fibrosis assessments. For future studies, more detailed analyses would be needed, such as molecular analyses to more assertively demonstrate the antifibrotic effects of these EVs in the CKD model.

In this study, the use of EVs from hUCT-derived MSCs and hUCB-derived eCD133^+^ cells prevent the progress of kidney damage in an adenine-induced renal injury model, which were demonstrated in albumin and serum cystatin C analyses, α-SMA labeling, and despite showing less significant expression only in the MSC-EV group, there was also an improvement in the eEPC-EV group.

The results obtained show that the current study may therefore contribute to developing approaches to improve the therapeutic efficacy of EVs isolated from hUCT-derived MSCs and hUCB-derived eCD133^+^ cells, since studies comparing treatments using EVs derived from two different cell types in the same disease and animal model are rare in the literature.

Furthermore, the present work can contribute to future studies, and it would be interesting to investigate the concomitant use of EVs from these two different cell types to treat CKD, since EV therapy may be an effective new approach to delay renal injury progression and improve renal function.

## 4. Materials and Methods

The procedures performed in this work were approved by the local Animal Research Ethics Committee and the Research Ethics Committee under numbers 0882 and CAAE 31647514.7.0000.0020, respectively. MSC samples were isolated from hUCT (single donor) and CD133^+^ cells were obtained from hUCB (single donor). Samples from both cellular sources were obtained after the presentation and signing of the informed consent form.

### 4.1. Isolation and Expansion of CD133^+^ Cells

hUCB was collected from the placenta while the umbilical cord was still attached. Blood collection was performed with syringes using the anticoagulant citric acid, sodium citrate and dextrose (JP Indústria Farmacêutica S.A., Ribeirão Preto, Brazil). The blood was placed in preprepared tubes with 10 milliliters of Iscove’s modified Dulbecco’s medium (IMDM) (Invitrogen, Auckland, New Zealand) supplemented with penicillin (100 μg/mL) and streptomycin (100 μg/mL) (Gibco™, Invitrogen, Grand Island, NY, USA).

MC isolation was performed according to the modified Boyum [85] method. For isolation, the cells were diluted 1:2 (*v*/*v*) in IMDM and centrifuged for 30 min at 400× g using Histopaque density gradients (Sigma–Aldrich, St. Louis, MO, USA). To purify CD 133^+^ cells, a CD133 microbead kit (Miltenyi Biotec^®^, Bergisch Gladbach, Germany) was used according to the manufacturer’s instructions. The purity of the cells was determined using anti-CD133 antibodies, and the viability of the cells was assessed by Annexin V and 7-AAD staining (BD Biosciences, San Diego, CA, USA). The samples were analyzed using a FACSCalibur flow cytometer (BD Biosciences). The isolated CD133^+^ cells were expanded as described by Senegaglia et al., 2010 [86]. EPCs were plated at a density of 1 × 10^5^ cells per cm^2^ in culture flasks and cultured in EBM-2 medium (endothelial cell basal medium) (Lonza Clonetics^®^, Walkersville, MD, USA) supplemented with 5% SBF, hydrocortisone, human fibroblast growth factor (hFGF), vascular endothelial growth factor (VEGF), insulin-like growth factor (IGF), ascorbic acid, and human epidermal growth factor (hEGF). The flasks were incubated at 37 °C with 5% CO_2_. Phenotypic characterization of expanded CD133^+^ cells was performed as previously described by our group [86].

### 4.2. Isolation and Expansion of MSCs

The isolation of hUCT-derived MSCs was performed according to a method that was previously described by our group [87], which consists of mechanical isolation, tissue maceration and enzymatic digestion with a 0.1% type II collagenase solution (Gibco™, Invitrogen, NY, USA) for 16 h at 37 °C with agitation followed by digestion with 0.25% trypsin/EDTA (Invitrogen™, Grand Island, NY, USA). The material was maintained for 15 min at 37 °C, and then, the enzymatic action was interrupted. The cells obtained at the end of this process were plated at a density of 1 × 10^5^ cells per cm^2^ in culture flasks (75 cm^2^, with filter cap) (TPP, Trasadingen, Switzerland) and cultured in IMDM supplemented with 15% fetal bovine serum (SBF-Gibco™, Invitrogen, NY, USA), 100 μg/mL streptomycin and 100 U/mL penicillin. The cells were incubated at 37 °C with 5% CO_2_. The culture medium was changed every 3–4 days until the cells reached confluence. When confluent, the adherent cells were detached using 0.25% trypsin-EDTA and replated at a concentration of 1.3 × 10^4^ cells per cm^2^ (first passage).

MSCs were characterized according to the markers as defined by the International Society for Cell & Gene Therapy (ISCT) [88]. The cells were incubated with the following monoclonal antibodies: anti-CD14, anti-CD19, and anti-CD45 antibodies conjugated to fluorescein isothiocyanate (FITC); anti-CD73, anti-CD90, and anti-CD166 antibodies conjugated to phycoerythrin (PE) antibodies; anti-HLA-DR conjugated to phycoerythrin-cyanine 5 PE-Cy5); and anti-CD29, anti-CD34, and anti-CD105 conjugated to allophycocyanin (APC). Additionally, cell viability was assessed by annexin PE and 7-AAD. Isotype controls for each fluorochrome were used in the reactions. All reagents were produced by BD Pharmingen (BD Biosciences, San Diego, CA, USA). The samples were acquired using a FACSCalibur flow cytometer and analyzed with FlowJo v8.0.2 software (Tree Star, Ashland, OR, USA).

### 4.3. Isolation of Extracellular Vesicles

EVs were isolated as previously described by Angulski et al., 2017 [17]. Briefly, after the cell cultures reached confluence, during the third–fourth passages, the cells were washed twice with phosphate saline (PBS—Gibco™, Invitrogen, NY, USA) and then cultured in their specific maintenance medium with vesicle-free 2% FBS to avoid cross-contamination. The conditioned medium of these cells was collected and submitted to a series of centrifugation steps: 700× *g* for 5 min to remove cellular debris; 4000× *g* for 20 min to remove apoptotic bodies; and two ultracentrifugation steps at 100,000× *g* for 70 min. The EV-enriched in pellet was quantified indirectly by measuring the protein content with a Qubit^®^ 2.0 Fluorometer (Life Technologies™, Invitrogen, NY, USA) and by direct analysis of suspended particles through nanoparticle tracking analysis with NanoSight (LM14C, Malvern Instruments). Vesicle morphology was determined by transmission electron microscopy as previously described by Angulski et al., 2017 [17]. After the isolation and characterization of EVs, they were stored at −80 °C until use.

### 4.4. Experimental Model of CKD & EV Infusions

For this study, 30 male Wistar rats (*Rattus norvegicus*) with an average weight of 280 g were used. The rats were housed in polypropylene cages (41 cm × 34 cm × 16 cm (height)) in groups of four rats/cage in a temperature (18–21 °C) and humidity-controlled environment (55–65% relative humidity) under a 12-h light-dark cycle and ad libitum access to standard rodent feed (NUVITAL^®^, Colombo, Paraná, Brazil) and water. The pine wood shavings (Inbrasfama, São José dos Pinhais, Paraná, Brazil) in each cage were changed daily. The animals underwent a ten-day acclimatization period before starting the experimental model.

**Figure 9:**
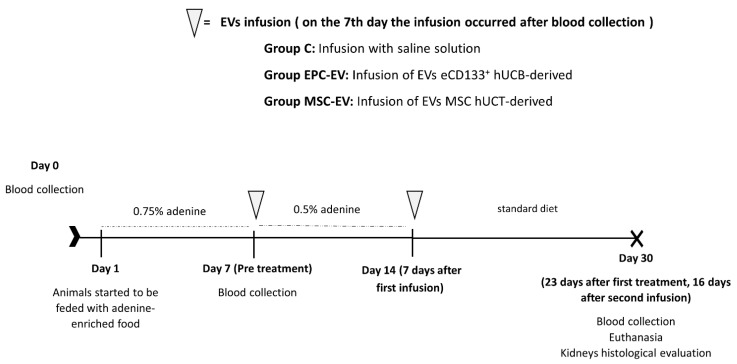
Study design chronic kidney disease induction using adenine and post treatment with extracellular vesicles from hUCT-derived MSCs and hUCB-derived eCD133^+^ cells in Wistar rats. Weighing and blood collection (Days 0, 07 and 30), extracellular vesicle infusion (Days 07 and 14), and euthanasia (Day 30) were performed.

The animal model of CKD induction using adenine was based on the work of Yokozawa et al., 1982 [16]. At the beginning of the study (Day 0), blood was collected from 30 animals (0.8 mL) through the jugular vein. On day one, the animals were placed in individual cages and given 15 g of pellet chow containing 0.75% adenine, and this was repeated for seven consecutive days. On the seventh day, blood was collected (0.8 mL) again, and ten animals were randomly chosen from each group to receive the first infusion of 30 μg/100 μL EVs derived from hUCT-derived MSCs (MSC-EV group), 30 μg/100 μL EVs derived from hUCB-derived eCD133^+^ cells (eEPC-EV group) and 100 μL of saline solution in Group C (control group) (Figure 9).

For the infusions, EVs were thawed at room temperature and homogenized on a shaker for one minute. EVs were prepared for infusion in a biological safety cabinet and placed in an insulin syringe (Benton and Dickinson, San Diego, CA, USA, Ultrafine, caliber 31G). Prior to the infusions, each animal’s tail was heated with a thermal bag to dilate the lateral vein of the tail and make it easier to identify, and the vein was garroted with the aid of an autostatic retractor.

After the first infusion, the animals received 15 g of pellet chow containing 0.5% adenine for seven consecutive days. On the 14th day, a second infusion of 30 μg/100 μL of EVs derived from hUCT-derived MSCs and hUCB-derived eCD133^+^ and saline solution was performed. From Day 14, the animals received a standard diet (Nuvilab^®^) until the end of the study. On the 30th day, blood was collected (0.8 mL), and euthanasia was performed by anesthetic overdose. Euthanasia was performed humanely, without the presence of other animals. Previously, after euthanasia, the rats were anesthetized with ketamine (100 mg/kg) and xylazine (10 mg/kg) and then exposed to an overdose of halothane after being placed in the induction chamber used to induce anesthesia. After the confirmation of death, each rat was necropsied (Figure 9).

### 4.5. Biochemical Analysis

Kidney function was analyzed by measuring serum creatinine (Cr), cystatin C (Cys C) and albumin. ELISA was used to measure serum cystatin C (Abcam^®^; Cat. No. ab201281, Cambridge, MA, USA) according to the kit instructions. Creatinine and albumin samples were sent for analysis at the Pontifical Catholic University of Paraná (PUCPR) veterinary hospital. Blood sample collection was performed prior to the induction of CKD and on the 7th and 30th days after the administration of adenine. The animals were anesthetized with 2.5% isoflurane, with an oxygen flow of 300 mL/min, according to the approximate time to perform the procedure.

### 4.6. Histopathological Analysis

H&E staining of renal tissue was performed to evaluate renal morphology, lesions, inflammatory processes, and the formation of 2.8-DHA crystals. Schiff’s periodic acid (PAS) staining was performed to evaluate connective tissue, and Picrosirius red staining (PRS) was performed to identify collagen deposition. The H&E and PAS slides were scanned with Axio Scan.Z1 (Zeiss, DE, Jena, Germany) and analyzed by Zen lite software. The PRS slides were analyzed using AxioScope.A1 microscopy and equipped with filters to provide polarized illumination.

### 4.7. Immunohistochemistry

Immunohistochemical evaluations were performed using α-SMA (Abcam, ab5694, 1:600 dilution, Cambridge, MA, USA) to identify myofibroblasts in the renal tissue and verify fibrosis and improvements after the treatments.

The slides were incubated at 55 °C for 10 min for deparaffinization in xylene and hydrated using a series of descending ethanol solutions. Subsequently, endogenous peroxidase was blocked, and antigen retrieval with an immuno retriever was performed for 25 min at 99 °C. The antibody (α-SMA) was then placed over the histological sections and incubated overnight at 4 °C. Labeling was revealed with the chromogen diaminobenzidine (DAB), and staining was performed with Harris hematoxylin. Subsequently, the sections were washed, dehydrated, cleared and covered with histological resin. The slides were scanned with Axio Scan.Z1 Zeiss and analyzed by Zen lite software.

### 4.8. Statistical Analysis

The results of the quantitative variables are described as the means, standard deviations, medians and ranges. Continuous variables that met the condition of normality in the three groups were compared using one-way analysis of variance (ANOVA) and the least significant test (LSD) for multiple comparisons. Other quantitative variables were analyzed using the nonparametric Kruskal–Wallis test. The evaluations within each group were compared with Student’s *t* test for paired samples or the nonparametric Wilcoxon test. The normality of the variables was assessed using the Shapiro–Wilk test. Values of *p* < 0.05 indicated statistical significance. Data were analyzed with the program IBM SPSS Statistics v.20.0 (IBM Corp., Armonk, NY, USA).

## Figures and Tables

**Table 1:** Serum albumin, creatinine and cystatin C evaluation. Evaluation of serum markers preinduction (Day 0) and at 7 days after CKD induction with adenine. A significant increase was observed in creatinine and cystatin C but not albumin. The data in the figures are reported as the mean ± sd. * *p* < 0.05 is considered significant.

Serum Markers	Evaluation Time	*n*	Mean	*p* *
SERUM ALBUMIN (g/dL)	Preinduction (Day 0)	27	4.51 ± 0.28	0.061
	Day 7	27	4.29 ± 0.63	
	Dif (Day 7–Day 0)	27	−0.22 ± 0.57	
SERUM CREATININE (mg/dL)	Preinduction (Day 0)	27	0.38 ± 0.09	0.001
	Day 7	27	0.58 ± 0.30	
	Dif (Day 7–Day 0)	27	0.20 ± 0.28	
CYSTATIN C	Preinduction (Day 0)	26	2498 ± 254	0.001
	Day 7	26	3269 ± 1054	
	Dif (Day 7–Day 0)	26	770.9 ± 1040.7	

## References

[1] Luyckx V.A., Tonelli M., Stanifer J.W. (2018). The Global Burden of Kidney Disease and the Sustainable Development Goals. Bull. World Health Organ..

[2] Filipska A., Bohdan B., Wieczorek P.P., Hudz N. (2021). Chronic Kidney Disease and Dialysis Therapy: Incidence and Prevalence in the World. Pharmacia.

[3] Li P.K.T., Garcia-Garcia G., Lui S.F., Andreoli S., Fung W.W.S., Hradsky A., Kumaraswami L., Liakopoulos V., Rakhimova Z., Saadi G. (2020). Kidney Health for Everyone Everywhere—From Prevention to Detection and Equitable Access to Care. Nefrologia.

[4] Romagnani P., Kalluri R. (2009). Possible Mechanisms of Kidney Repair. Fibrogenes. Tissue Repair.

[5] Peired A.J., Sisti A., Romagnani P. (2016). Mesenchymal Stem Cell-Based Therapy for Kidney Disease: A Review of Clinical Evidence. Stem Cells Int..

[6] Karpman D., Ståhl A.L., Arvidsson I. (2017). Extracellular Vesicles in Renal Disease. Nat. Rev. Nephrol..

[7] Ozkok A., Yildiz A. (2018). Endothelial Progenitor Cells and Kidney Diseases. Kidney Blood Press. Res..

[8] Asahara T., Murohara T., Sullivan A., Silver M., Van Der Zee R., Li T., Witzenbichler B., Schatteman G., Isner J.M. (1997). Isolation of Putative Progenitor Endothelial Cells for Angiogenesis. Science.

[9] Spees J.L., Lee R.H., Gregory C.A. (2016). Mechanisms of Mesenchymal Stem/Stromal Cell Function. Stem Cell Res. Ther..

[10] Bunnell B.A., Betancourt A.M., Sullivan D.E., Barry F.P., Murphy J.M. (2004). Mesenchymal Stem Cells: Clinical Applications and Biological Characterization. Int. J. Biochem. Cell Biol..

[11] Altabas V., Altabas K., Kirigin L. (2016). Endothelial Progenitor Cells (EPCs) in Ageing and Age-Related Diseases: How Currently Available Treatment Modalities Affect EPC Biology, Atherosclerosis, and Cardiovascular Outcomes. Mech. Ageing Dev..

[12] Gatti S., Bruno S., Deregibus M.C., Sordi A., Cantaluppi V., Tetta C., Camussi G. (2011). Microvesicles Derived from Human Adult Mesenchymal Stem Cells Protect against Ischaemia-Reperfusion-Induced Acute and Chronic Kidney Injury. Nephrol. Dial. Transplant..

[13] Cantaluppi V., Gatti S., Medica D., Figliolini F., Bruno S., Deregibus M.C., Sordi A., Biancone L., Tetta C., Camussi G. (2012). Microvesicles Derived from Endothelial Progenitor Cells Protect the Kidney from Ischemia-Reperfusion Injury by MicroRNA-Dependent Reprogramming of Resident Renal Cells. Kidney Int..

[14] Bian S., Zhang L., Duan L., Wang X., Min Y., Yu H. (2014). Extracellular Vesicles Derived from Human Bone Marrow Mesenchymal Stem Cells Promote Angiogenesis in a Rat Myocardial Infarction Model. J. Mol. Med..

[15] He J., Wang Y., Sun S., Yu M., Wang C., Pei X., Zhu B., Wu J., Zhao W. (2012). Bone Marrow Stem Cells-Derived Microvesicles Protect against Renal Injury in the Mouse Remnant Kidney Model. Nephrology.

[16] Oura H. (1982). Takako of Dietary Purine in Rats, Faculty of Medicine, Toyama Medical and Pharmaceutical University, Were as Follows: Hypoxaiithine, Ular, the Intake of Adenine Exhibited Previously Published Data from Our Laboratory on Measuring the Level of Uric A. J. Nutr. Sci. Vitaminol..

[17] Angulski A.B.B., Capriglione L.G., Batista M., Marcon B.H., Senegaglia A.C., Stimamiglio M.A., Correa A. (2017). The Protein Content of Extracellular Vesicles Derived from Expanded Human Umbilical Cord Blood-Derived CD133^+^ and Human Bone Marrow-Derived Mesenchymal Stem Cells Partially Explains Why Both Sources Are Advantageous for Regenerative Medicine. Stem Cell Rev. Rep..

[18] Claramunt D., Gil-Peña H., Fuente R., Hernández-Frías O., Santos F. (2015). Animal Models of Pediatric Chronic Kidney Disease. Is Adenine Intake an Appropriate Model?. Nefrologia.

[19] Engle S.J., Stockelmant M.G., Chen J.U., Boivint G., Yum M., Daviest P.M., Yingt M.O.Y.I.N., Sahota A., Simmondst H.A., Stambrookt P.J. (1996). Develop 2,8-Dihydroxyadenine. Genetics.

[20] Tögel F., Hu Z., Weiss K., Isaac J., Lange C., Westenfelder C. (2005). Administered Mesenchymal Stem Cells Protect against Ischemic Acute Renal Failure through Differentiation-Independent Mechanisms. Am. J. Physiol.-Ren. Physiol..

[21] Sun Y.Q., Deng M.X., He J., Zeng Q.X., Wen W., Wong D.S.H., Tse H.F., Xu G., Lian Q., Shi J. (2012). Human Pluripotent Stem Cell-Derived Mesenchymal Stem Cells Prevent Allergic Airway Inflammation in Mice. Stem Cells.

[22] Suss P.H., Capriglione L.G.A., Barchiki F., Miyague L., Jackowski D., Fracaro L., Schittini A.V., Senegaglia A.C., Rebelatto C.L.K., Olandoski M. (2015). Direct Intracardiac Injection of Umbilical Cord-Derived Stromal Cells and Umbilical Cord Blood-Derived Endothelial Cells for the Treatment of Ischemic Cardiomyopathy. Exp. Biol. Med..

[23] Correa A., Ottoboni G.S., Senegaglia A.C., Capriglione L.G.A., Miyague N.I., Leite L.M.B., Jamur V.R., Rebelatto C.L.K., Olandoski M., Brofman P.R.S. (2018). Expanded CD133^+^ Cells from Human Umbilical Cord Blood Improved Heart Function in Rats after Severe Myocardial Infarction. Stem Cells Int..

[24] Boldrini-Leite L.M., Michelotto P.V., de Moura S.A.B., Capriglione L.G.A., Barussi F.C.M., Fragoso F.Y.I., Senegaglia A.C., Brofman P.R.S. (2020). Lung Tissue Damage Associated with Allergic Asthma in BALB/c Mice Could Be Controlled with a Single Injection of Mesenchymal Stem Cells from Human Bone Marrow up to 14 d After Transplantation. Cell Transplant..

[25] Bruno S., Grange C., Collino F., Deregibus M.C., Cantaluppi V., Biancone L., Tetta C., Camussi G. (2012). Microvesicles Derived from Mesenchymal Stem Cells Enhance Survival in a Lethal Model of Acute Kidney Injury. PLoS ONE.

[26] Lamichhane T.N., Sokic S., Schardt J.S., Raiker R.S., Lin J.W., Jay S.M. (2015). Emerging Roles for Extracellular Vesicles in Tissue Engineering and Regenerative Medicine. Tissue Eng.-Part B Rev..

[27] Sun X., Meng H., Wan W., Xie M., Wen C. (2019). Application Potential of Stem/Progenitor Cell-Derived Extracellular Vesicles in Renal Diseases. Stem Cell Res. Ther..

[28] Momen-Heravi F., Balaj L., Alian S., Tigges J., Toxavidis V., Ericsson M., Distel R.J., Ivanov A.R., Skog J., Kuo W.P. (2012). Alternative Methods for Characterization of Extracellular Vesicles. Front. Physiol..

[29] Van der Pol E., Böing A.N., Harrison P., Sturk A., Nieuwland R. (2012). Classification, Functions, and Clinical Relevance of Extracellular Vesicles. Pharmacol. Rev..

[30] Ali B.H., Ziada A., Al Husseni I., Beegam S., Nemmar A. (2011). Motor and Behavioral Changes in Rats with Adenine-Induced Chronic Renal Failure: Influence of Acacia Gum Treatment. Exp. Biol. Med..

[31] Al Suleimani Y., Al M., Ramkumar A., Almahruqi A. (2015). Influence of treatment with gum acacia on renal vascular responses in a rat model of chronic kidney disease. Eur. Rev. Med. Pharmacol. Sci..

[32] Diwan V., Brown L., Gobe G.C. (2018). Adenine-Induced Chronic Kidney Disease in Rats. Nephrology.

[33] Li Q.M., Chena H.R., Zha X.Q., Lu C.Q., Pan L.H., Luo J.P. (2018). Renoprotective Effect of Chinese Chive Polysaccharides in Adenine-Induced Chronic Renal Failure. Int. J. Biol. Macromol..

[34] Lou X., Jin J., Gong J., Zhao L., Li Y., He Q. (2019). Comparison of the Effects of Indobufen and Warfarin in a Rat Model of Adenine-Induced Chronic Kidney Disease. Med. Sci. Monit..

[35] Friedman A.N., Fadem S.Z. (2010). Reassessment of Albumin as a Nutritional Marker in Kidney Disease. J. Am. Soc. Nephrol..

[36] Rivera-Valdés J.J., García-Bañuelos J., Salazar-Montes A., García-Benavides L., Rosales-Dominguez A., Armendáriz-Borunda J., Sandoval-Rodríguez A. (2017). Human Adipose Derived Stem Cells Regress Fibrosis in a Chronic Renal Fibrotic Model Induced by Adenine. PLoS ONE.

[37] Do Carmo W.B., Castro B.B.A., Rodrigues C.A., Custódio M.R., Sanders-Pinheiro H. (2018). Chitosan-Fe (III) Complex as a Phosphate Chelator in Uraemic Rats: A Novel Treatment Option. Basic Clin. Pharmacol. Toxicol..

[38] Ali B.H., Al Za’abi M., Adham S.A., Al Suleimani Y., Karaca T., Manoj P., Al Kalbani J., Yasin J., Nemmar A. (2018). The Effect of Sildenafil on Rats with Adenine—Induced Chronic Kidney Disease. Biomed. Pharmacother..

[39] Thakur R., Sharma A., Lingaraju M.C., Begum J., Kumar D., Mathesh K., Kumar P., Singh T.U., Kumar D. (2018). Ameliorative Effect of Ursolic Acid on Renal Fibrosis in Adenine-Induced Chronic Kidney Disease in Rats. Biomed. Pharmacother..

[40] Dos Santos N.S.J., Draibe S.A., Kamimura M.A., Cuppari L. (2004). Albumina Sérica Como Marcador Nutricional de Pacientes Em Hemodiálise. Rev. Nutr..

[41] Tynkevich E., Flamant M., Haymann J.P., Metzger M., Thervet E., Boffa J.J., Vrtovsnik F., Houillier P., Froissart M., Stengel B.N.D. (2014). Decrease in Urinary Creatinine Excretion in Early Stage Chronic Kidney Disease. PLoS ONE.

[42] Okabe C., Borges R.L., de Almeida D.C., Fanelli C., Barlette G.P., Machado F.G., Arias S.C.A., Malheiros D.M.A.C., Camara N.O.S., Zatz R. (2013). NF-ΚB Activation Mediates Crystal Translocation and Interstitial Inflammation in Adenine Overload Nephropathy. Am. J. Physiol.-Ren. Physiol..

[43] Stevens L.A., Coresh J., Greene T., Levey A.S. (2006). Assessing Kidney Function—Measured and Estimated Glomerular Filtration Rate. N. Engl. J. Med..

[44] Peralta C.A., Shlipak M.G., Judd S., Cushman M., McClellan W., Zakai N.A., Safford M.M., Zhang X., Muntner P., Warnock D. (2011). Detection of Chronic Kidney Disease With Creatinine, Cystatin C, and Urine Albumin-to-Creatinine Ratio and Association With Progression to End-Stage Renal Disease and Mortality. JAMA.

[45] Vaidya V.S., Ferguson M.A., Bonventre J.V. (2008). Biomarkers of Acute Kidney Injury. Annu. Rev. Pharmacol. Toxicol..

[46] Shemesh O., Golbetz H., Kriss J.P., Myers B.D. (1985). Limitations of Creatinine as a Filtration Marker in Glomerulopathic Patients. Kidney Int..

[47] Zou L.X., Sun L., Nicholas S.B., Lu Y., K S.S., Hua R. (2020). Comparison of Bias and Accuracy Using Cystatin C and Creatinine in CKD-EPI Equations for GFR Estimation. Eur. J. Intern. Med..

[48] Murty M.S.N., Sharma U.K., Pandey V.B., Kankare S.B. (2013). Serum Cystatin C as a Marker of Renal Function in Detection of Early Acute Kidney Injury. Indian J. Nephrol..

[49] Benoit S.W., Ciccia E.A., Devarajan P. (2020). Cystatin C as a Biomarker of Chronic Kidney Disease: Latest Developments. Expert Rev. Mol. Diagn..

[50] Gharaibeh K.A., Hamadah A.M., El-Zoghby Z.M., Lieske J.C., Larson T.S., Leung N. (2018). Cystatin C Predicts Renal Recovery Earlier Than Creatinine Among Patients With Acute Kidney Injury. Kidney Int. Rep..

[51] Diwan V., Mistry A., Gobe G., Brown L. (2013). Adenine-Induced Chronic Kidney and Cardiovascular Damage in Rats. J. Pharmacol. Toxicol. Methods.

[52] Törmänen S., Pörsti I., Lakkisto P., Tikkanen I., Niemelä O., Paavonen T., Mustonen J., Eräranta A. (2017). Endothelin A Receptor Blocker and Calcimimetic in the Adenine Rat Model of Chronic Renal Insufficiency. BMC Nephrol..

[53] Puthumana J., Thiessen-Philbrook H., Xu L., Coca S.G., Garg A.X., Himmelfarb J., Bhatraju P.K., Ikizler T.A., Siew E.D., Ware L.B. (2021). Biomarkers of Inflammation and Repair in Kidney Disease Progression. J. Clin. Investig..

[54] Chevalier R.L., Forbes M.S., Thornhill B.A. (2009). Ureteral Obstruction as a Model of Renal Interstitial Fibrosis and Obstructive Nephropathy. Kidney Int..

[55] Meng X., Nikolic-paterson D.J., Lan H.Y. (2014). Inflammatory Processes in Renal Fibrosis. Nat. Publ. Gr..

[56] Distler J.H.W., Györfi A.H., Ramanujam M., Whitfield M.L., Königshoff M., Lafyatis R. (2019). Shared and Distinct Mechanisms of Fibrosis. Nat. Rev. Rheumatol..

[57] Djudjaj S., Boor P. (2019). Cellular and Molecular Mechanisms of Kidney Fibrosis. Mol. Asp. Med..

[58] Panizo S., Martínez-Arias L., Alonso-Montes C., Cannata P., Martín-Carro B., Fernández-Martín J.L., Naves-Díaz M., Carrillo-López N., Cannata-Andía J.B. (2021). Fibrosis in Chronic Kidney Disease: Pathogenesis and Consequences. Int. J. Mol. Sci..

[59] Meran S., Steadman R. (2011). Fibroblasts and Myofibroblasts in Renal Fibrosis. Int. J. Exp. Pathol..

[60] Rodrigues P.C., Da Costa Miguel M.C., De Aquino S.N., Fonseca F.P., Dos Santos Silva A.R., Leme A.F.P., Coletta R.D. (2015). Stromal Myofibroblasts in Potentially Malignant and Malignant Lesions of the Oral Cavity. Oncol. Lett..

[61] Orimo A., Sato M., Nogi Y., Inoue S., Muramatsu M., Tomioka Y., Oigawa S., Hata T., Shimizu Y., Kamata K. (2001). Cancer-Associated Myofibroblasts Possess Various Factors to Promote Endometrial Tumor Progression. Clin. Cancer Res..

[62] Koyama Y., Brenner D.A. (2017). Liver Inflammation and Fibrosis. J. Clin. Investig..

[63] Lepreux S., Desmoulière A. (2015). Human Liver Myofibroblasts during Development and Diseases with a Focus on Portal (Myo)Fibroblasts. Front. Physiol..

[64] Dees C., Chakraborty D., Distler J.H.W. (2021). Cellular and Molecular Mechanisms in Fibrosis. Exp. Dermatol..

[65] Yuan Q., Tan R.J., Liu Y. (2019). Myofibroblast in Kidney Fibrosis: Origin, Activation, and Regulation.

[66] Isaka Y. (2018). Targeting TGF-β Signaling in Kidney Fibrosis. Int. J. Mol. Sci..

[67] Eardley K.S., Kubal C., Zehnder D., Quinkler M., Lepenies J., Savage C.O., Howie A.J., Kaur K., Cooper M.S., Adu D. (2008). The Role of Capillary Density, Macrophage Infiltration and Interstitial Scarring in the Pathogenesis of Human Chronic Kidney Disease. Kidney Int..

[68] Rodrigues C.E., Capcha J.M.C., De Bragança A.C., Sanches T.R., Gouveia P.Q., De Oliveira P.A.F., Malheiros D.M.A.C., Volpini R.A., Santinho M.A.R., Santana B.A.A. (2017). Human Umbilical Cord-Derived Mesenchymal Stromal Cells Protect against Premature Renal Senescence Resulting from Oxidative Stress in Rats with Acute Kidney Injury. Stem Cell Res. Ther..

[69] Du T., Cheng J., Zhong L., Zhao X.F., Zhu J., Zhu Y.J., Liu G.H. (2012). The Alleviation of Acute and Chronic Kidney Injury by Human Wharton’s Jelly-Derived Mesenchymal Stromal Cells Triggered by Ischemia-Reperfusion Injury via an Endocrine Mechanism. Cytotherapy.

[70] Zhang G., Zou X., Miao S., Chen J., Du T., Zhong L., Ju G., Liu G., Zhu Y. (2014). The Anti-Oxidative Role of Micro-Vesicles Derived from Human Wharton-Jelly Mesenchymal Stromal Cells through NOX2/Gp91(Phox) Suppression in Alleviating Renal Ischemia-Reperfusion Injury in Rats. PLoS ONE.

[71] Wu X., Yan T., Wang Z., Wu X., Cao G., Zhang C., Tian X., Wang J. (2018). Micro-Vesicles Derived from Human Wharton’s Jelly Mesenchymal Stromal Cells Mitigate Renal Ischemia-Reperfusion Injury in Rats after Cardiac Death Renal Transplantation. J. Cell. Biochem..

[72] Zou X., Zhang G., Cheng Z., Yin D., Du T., Ju G., Miao S., Liu G., Lu M., Zhu Y. (2014). Microvesicles Derived from Human Wharton’s Jelly Mesenchymal Stromal Cells Ameliorate Renal Ischemia-Reperfusion Injury in Rats by Suppressing CX3CL1. Stem Cell Res. Ther..

[73] Barnes J.L., Gorin Y. (2011). Myofibroblast Differentiation During Fibrosis: Role of NAD(P)H Oxidases. Kidney Int..

[74] Herrera M.B., Bussolati B., Bruno S., Fonsato V., Romanazzi G.M., Camussi G. (2004). Mesenchymal Stem Cells Contribute to the Renal Repair of Acute Tubular Epithelial Injury. Int. J. Mol. Med..

[75] Lange C., Tögel F., Ittrich H., Clayton F., Nolte-Ernsting C., Zander A.R., Westenfelder C. (2005). Administered Mesenchymal Stem Cells Enhance Recovery from Ischemia/Reperfusion-Induced Acute Renal Failure in Rats. Kidney Int..

[76] Morigi M., Introna M., Imberti B., Corna D., Abbate M., Rota C., Rottoli D., Benigni A., Perico N., Zoja C. (2008). Human Bone Marrow Mesenchymal Stem Cells Accelerate Recovery of Acute Renal Injury and Prolong Survival in Mice. Stem Cells.

[77] Chade A.R., Zhu X., Lavi R., Krier J.D., Pislaru S., Simari R.D., Napoli C., Lerman A., Lerman L.O. (2009). Endothelial Progenitor Cells Restore Renal Function in Chronic Experimental Renovascular Disease. Circulation.

[78] Eirin A., Lerman L.O. (2014). Mesenchymal Stem Cell Treatment for Chronic Renal Failure. Stem Cell Res. Ther..

[79] Semedo P., Correa-Costa M., Cenedeze M.A., Malheiros D.M.A.C., Dos Reis M.A., Shimizu M.H., Seguro A.C., Pacheco-Silva A., Ĉamara N.O.S. (2009). Mesenchymal Stem Cells Attenuate Renal Fibrosis through Immune Modulation and Remodeling Properties in a Rat Remnant Kidney Model. Stem Cells.

[80] Tögel F., Weiss K., Yang Y., Hu Z., Zhang P., Westenfelder C. (2007). Vasculotropic, Paracrine Actions of Infused Mesenchymal Stem Cells Are Important to the Recovery from Acute Kidney Injury. Am. J. Physiol.-Ren. Physiol..

[81] De Jong O.G., van Balkom B.W.M., Schiffelers R.M., Bouten C.V.C., Verhaar M.C. (2014). Extracellular Vesicles: Potential Roles in Regenerative Medicine. Front. Immunol..

[82] Nassar W., El-Ansary M., Sabry D., Mostafa M.A., Fayad T., Kotb E., Temraz M., Saad A.N., Essa W., Adel H. (2016). Umbilical Cord Mesenchymal Stem Cells Derived Extracellular Vesicles Can Safely Ameliorate the Progression of Chronic Kidney Diseases. Biomater. Res..

[83] Kao M.P., Ang D.S., Pall A., Struthers A.D. (2010). Oxidative Stress in Renal Dysfunction: Mechanisms, Clinical Sequelae and Therapeutic Options. J. Hum. Hypertens..

[84] Xia J., Swiercz J.M., Bañón-Rodríguez I., Matković I., Federico G., Sun T., Franz T., Brakebusch C.H., Kumanogoh A., Friedel R.H. (2015). Semaphorin-Plexin Signaling Controls Mitotic Spindle Orientation during Epithelial Morphogenesis and Repair. Dev. Cell.

[85] Bøyum A. (1968). Isolation of Mononuclear Cells and Granulocytes from Human Blood. Scand. J. Clin. Lab. Investig..

[86] Senegaglia A.C., Barboza L.A., Dallagiovanna B., Aita C.A.M., Hansen P., Rebelatto C.L.K., Aguiar A.M., Miyague N.I., Barchiki F., Correa A. (2010). Better at Improving Cardiac Function?. Exp. Biol. Med..

[87] Rebelatto C.K., Aguiar A.M., Moretão M.P., Senegaglia A.C., Hansen P., Barchiki F., Oliveira J., Martins J., Kuligovski C., Mansur F. (2008). Dissimilar Differentiation of Mesenchymal Stem Cells from Bone Marrow, Umbilical Cord Blood, and Adipose Tissue. Exp. Biol. Med..

[88] Dominici M., Le Blanc K., Mueller I., Slaper-Cortenbach I., Marini F.C., Krause D.S., Deans R.J., Keating A., Prockop D.J., Horwitz E.M. (2006). Minimal Criteria for Defining Multipotent Mesenchymal Stromal Cells. The International Society for Cellular Therapy Position Statement. Cytotherapy.

